# Computer-aided recognition of myopic tilted optic disc using deep learning algorithms in fundus photography

**DOI:** 10.1186/s12886-020-01657-w

**Published:** 2020-10-09

**Authors:** Baek Hwan Cho, Da Young Lee, Kyung-Ah Park, Sei Yeul Oh, Jong Hak Moon, Ga-In Lee, Hoon Noh, Joon Kyo Chung, Min Chae Kang, Myung Jin Chung

**Affiliations:** 1 Medical AI Research Center, Institute of Smart Healthcare, Samsung Medical Center, Seoul, Korea; 2 Department of Medical Device Management and Research, SAIHST, Sungkyunkwan University, Seoul, Korea; 3 Department of Digital Health, SAIHST, Sungkyunkwan University, Seoul, Korea; 4 Department of Ophthalmology, Samsung Medical Center, Sungkyunkwan University School of Medicine, 81 Irwon-ro, Gangnam-gu, Seoul, 06351 Korea; 5 Department of Radiology, Samsung Medical Center, Sungkyunkwan University School of Medicine, Seoul, Korea

**Keywords:** Fundus photographs, Myopic tilted optic disc, Deep learning algorithm, Automated computer-aided recognition

## Abstract

**Background:**

It is necessary to consider myopic optic disc tilt as it seriously impacts normal ocular parameters. However, ophthalmologic measurements are within inter-observer variability and time-consuming to get. This study aimed to develop and evaluate deep learning models that automatically recognize a myopic tilted optic disc in fundus photography.

**Methods:**

This study used 937 fundus photographs of patients with normal or myopic tilted disc, collected from Samsung Medical Center between April 2016 and December 2018. We developed an automated computer-aided recognition system for optic disc tilt on color fundus photographs via a deep learning algorithm. We preprocessed all images with two image resizing techniques. GoogleNet Inception-v3 architecture was implemented. The performances of the models were compared with the human examiner’s results. Activation map visualization was qualitatively analyzed using the generalized visualization technique based on gradient-weighted class activation mapping (Grad-CAM++).

**Results:**

Nine hundred thirty-seven fundus images were collected and annotated from 509 subjects. In total, 397 images from eyes with tilted optic discs and 540 images from eyes with non-tilted optic discs were analyzed. We included both eye data of most included patients and analyzed them separately in this study. For comparison, we conducted training using two aspect ratios: the simple resized dataset and the original aspect ratio (AR) preserving dataset, and the impacts of the augmentations for both datasets were evaluated. The constructed deep learning models for myopic optic disc tilt achieved the best results when simple image-resizing and augmentation were used. The results were associated with an area under the receiver operating characteristic curve (AUC) of 0.978 ± 0.008, an accuracy of 0.960 ± 0.010, sensitivity of 0.937 ± 0.023, and specificity of 0.963 ± 0.015. The heatmaps revealed that the model could effectively identify the locations of the optic discs, the superior retinal vascular arcades, and the retinal maculae.

**Conclusions:**

We developed an automated deep learning-based system to detect optic disc tilt. The model demonstrated excellent agreement with the previous clinical criteria, and the results are promising for developing future programs to adjust and identify the effect of optic disc tilt on ophthalmic measurements.

## Background

Tilted discs can be classified into two groups based on etiology: congenital tilted disc syndrome, which is an anomaly of the eye and is characterized by inferior or inferonasal tilting of the optic disc [1, 2], and myopic tilted disc, which is an acquired change related to progression of myopia [1]. Previous studies have illustrated optic disc tilt development and temporal crescent formation over time, and disc tilting develops in the relatively early stages of mild myopia in some patients.

The prevalence of myopia has increased and is expected to continue increasing globally [3]. The expected population of individuals affected by myopia is reported to be 4758 million by 2050 [3]. The prevalence is significantly higher in countries of the Asia-Pacific region compared with other regions and is dramatically increasing in East Asia [3, 4]. Consequently, the clinical significance of myopic optic disc tilt might also be increasing.

Optic disc tilt can lead to significant changes in optic disc appearance [5–7] and affects ocular parameters used in the majority of ophthalmic devices, such as optical coherence tomography [8, 9] and visual field analyzers [10–12], which are the most widely used devices in ophthalmology. However, it is difficult to obtain normal measurement results for patients with tilted optic discs using most ophthalmologic instruments, and the ophthalmic measurements in these patients are interpreted based on the supervising physician’s discretion.

Medical image analysis that uses deep learning algorithms has recently gained attention due to the variety of technological applications, including image recognition and speech recognition, as well as medical applications [13, 14]. Numerous studies have used deep learning algorithms to characterize and diagnose several diseases from fundus images [15–18]. However, to the best of our knowledge, there is limited research that has focused on tilted optic discs using deep learning, even though it is significant for ophthalmologic diagnosis systems or disease progression recognition systems.

To construct an ophthalmologic automatic diagnosis system or disease progression recognition system, it is necessary to consider myopic optic disc tilt as it seriously impacts normal ophthalmological measurements. This will be of greater clinical significance with the increasing population of myopic patients. The first step in developing a technology for a fully automated diagnosis system is to automatically recognize the presence of a tilted disc. This can be the basis of an automated clinical decision support system that enables calibration of the tilted disc to distinguish abnormal from normal ocular measurements.

Thus, this study aimed to develop a fully automated system for detecting tilted disc in fundus photographs using deep learning algorithms. This system can provide a framework for deep learning-based research that is focused on other tilted disc-related diseases. We evaluated the algorithm’s ability to differentiate between subjects with from those without tilted optic discs under various experimental settings. Activation map visualizations are also provided and show which parts of the fundus images are related to the algorithm decision process.

## Methods

This study was performed at a single center and was performed in accordance with the tenets of the Declaration of Helsinki. This study was approved by the Institutional Review Board of Samsung Medical Center (Seoul, Republic of Korea, Approval No.: 2018–11-018). Informed consent was waived for the patients in this study.

### Patients

Nine hundred thirty-seven fundus photographs of normal patients and patients with myopic tilted discs were collected from Samsung Medical Center between April 2016 and December 2018. Fundus photographs were acquired using a TRC-50IX digital camera (Topcon, Tokyo, Japan) or Kowa nonmyd 10 megapixel fundus camera (Kowa, Torrance, CA). Enrollment criteria for myopic tilted discs were as follows: an optic disc with a ratio of minimal to maximal disc diameter of 0.75 or less on the fundus photograph, as described in previous studies [19, 20], a white semilunar patch of sclera adjacent to the optic disc [21–23], and − 0.5 diopters (D) or more of myopia.

Only temporally tilted discs were considered myopic tilted discs, and discs tilted in another direction, including nasally, superiorly, or inferiorly, were excluded to avoid including tilted discs with a congenital etiology. Tilted discs with axes beyond 45 degrees of the vertical meridian were also excluded. Normal controls had normal optic disc shapes without semilunar patches of sclera adjacent to the optic disc.

**Fig. 1:**
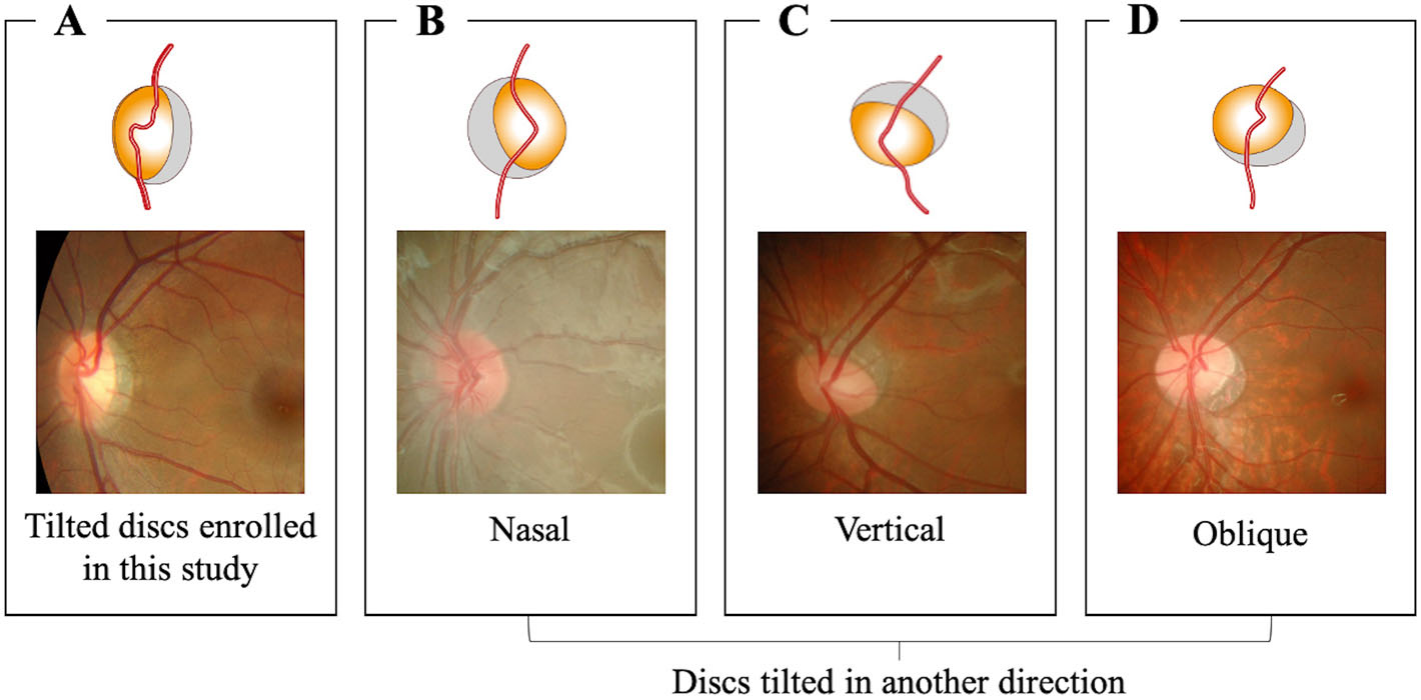
Illustration that shows the included and the excluded anatomical findings of the optic disc. **a**, a representative myopic tilted optic disc (included in the study); **b**, nasally tilted disc (excluded from the study); **c**, vertically tilted disc (excluded from the study); **d**, obliquely tilted disc (excluded from the study)

Fundus photographs with poor image quality that could not be used for analysis were also excluded. Exclusion criteria for both patients with myopic tilted discs and healthy controls included previous eye trauma or eye surgery and any ocular pathology that may affect fundus photography, with the exception for refractive error. Patient demographics and refractive data were collected from medical records. Figure 1 is an illustration of included and excluded anatomical findings of the optic disc.

### Data preprocessing

The original dataset included various image sizes because of different device settings. Several images were of both eyes in one photograph; therefore, we split those images so that each side represented one of the eyes. Some of the original images also contained text information about the patients; this information was cropped to optimize extraction.

To obtain a fixed size of an input image for training, we resized all the images to a resolution of 524 × 400 pixels. However, such image resizing could lead to distortion of the aspect ratio (AR). Inevitably, in regions of the optic disc, locations of objects in the fundus could be distorted. Because the angle between the disc and the other retinal sections is correlated with disc tilt angle, simple image resizing could affect deep learning model training [24–26]. Consequently, another preprocessing method was applied to resize images to try and preserve the AR of the original images. The image preprocessing for the AR-preserving dataset included the following steps: (1) crop the black borders of both the left and right sides from the fundus images to fit the exact area of the retinal fundus; (2) calculate the AR distribution of the border-cropped dataset; (3) select the most common aspect ratio from the distribution; (4) preserve the original height of each image and either crop or zero-pad the left and right sides to establish it as the selected AR; (5) resize each image to a resolution of 524 × 436 pixels for the AR-preserving dataset.

**Fig. 2:**
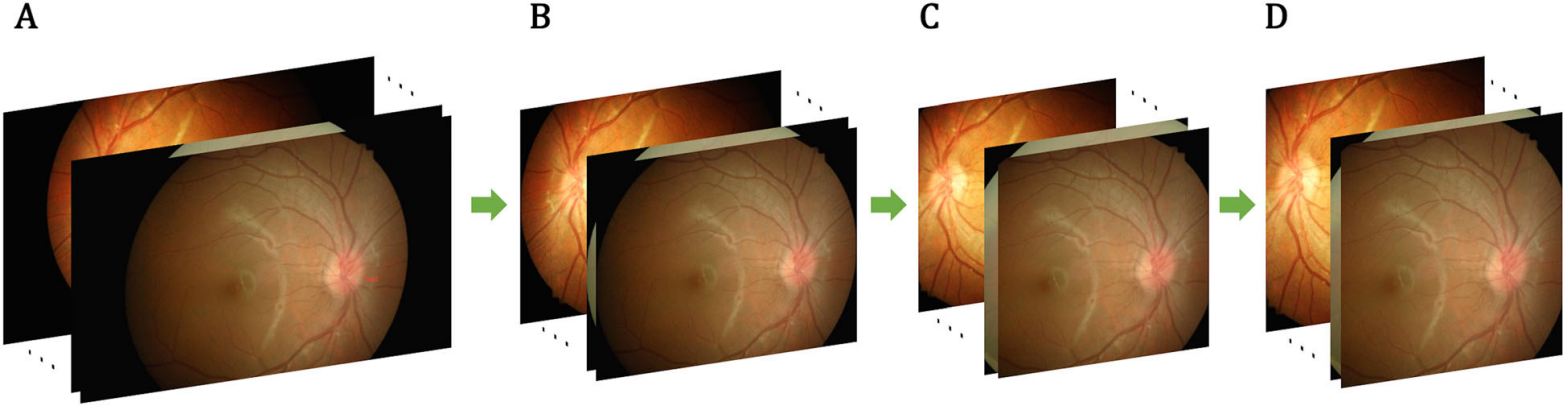
Overview of data preprocessing for the AR-preserving dataset. **a**, data cleansing; **b**, cropping the black border area and determining the aspect ratio distribution of the actual fundus area; **c**, cropping or zero-padding while keeping the height intact; **d**, resizing each image to a resolution of 524 X 436

Figure 2 is an overview of data preprocessing for the AR-preserving dataset. Steps (1)–(3) above are illustrated in Fig. 2b. The original fundus images are composed of the actual fundus portion and the black border on both the left and right sides. Thus, we implemented a method that automatically crops the black background on both sides based on the intensity of the middle row of each image because the background has low intensity. Figure 2c illustrates step (4) to place the background-cropped image into the template. Finally, we resized the template image by preserving the AR of the original image, as in Fig. 2d.

### Deep learning model training

In this study, we used K-fold stratified cross-validation [27, 28] to evaluate the performance accuracy of our proposed model so that each fold has the same distributions of classes as the whole dataset.

The preprocessed input dataset is divided into K non-overlapping subsets balancing the number of images per class. K-1 subsets are used for training, and the remaining set is used for validation. This process is repeated for all K partitions. There were several cases for which we collected multiple fundus images from a single subject. Those cases had inconsistent clinical imaging findings since we collected images from both eyes and on different dates. We split the dataset into 5 subsets with respect to the patient IDs so that data with the same ID would not be distributed to both training and test sets. The dataset was split patient-wise according to the proportion of 0.6:0.4 for normal and abnormal class across each fold.

The images of the training dataset were augmented by mirror flipping, brightness control, and intensity control to enhance the size and quality. Since regions in the fundus have bilateral symmetry, we flipped some images horizontally. We also assigned lighting bias with a value between 0.8 and 1.2 and increased the pixel values in range of 0–4. Even though these transformations are among the most common for image classification problems, we cannot ensure that the labels of the original images are not altered. Therefore, augmentation options had to be carefully estimated, and the generated images were observed by clinicians manually. Consequently, the amount of training data was increased by 50 times after augmentation.

The input dataset included RGB images with a range of 0–255 pixel values for each RGB channel. We used global centering for the datasets by calculating and subtracting the mean values per pixel of the training data across all the color channels. Pixel-wise mean subtraction allowed the distribution of pixel values to be centered at zero [29, 30]. Next, each channel was normalized to the range of 0–1 [31, 32]. Both the simple image resized dataset and the original AR preserved dataset were prepared following the procedures described above.

GoogleNet Inception-v3 architecture was implemented as a base model [33]. The model was initialized by the ImageNet pre-trained model [34, 35] and fine-tuned with our datasets [36–38]. The weights of the pre-trained model were used to initialize each layer of the model and were updated as training proceeded. The performance of this algorithm was compared with that of a human examiner, who is an expert in identifying myopic disc appearance. Areas under the receiver operating characteristic curves, sensitivity, and specificity were computed for each of the models [39–41].

## Results

Nine hundred thirty-seven fundus images were collected and annotated. Among those, 397 images were from eyes with tilted optic discs and 540 images were from eyes with non-tilted optic discs. A total of 509 subjects participated in this study. The mean age in the cases with myopic tilted disc was 10.8 (standard deviation, SD = 3.5; range, 1–55) years, and that in normal cases was 10.3 (SD = 5.2; range, 2–70) years. There was no statistical difference in age between the two groups (*p* = 0.45). Mean spherical equivalent refraction (SER) of the cases with tilted optic discs was - 5.57D ± 3.74D in the range of − 19.5 to 3.5D, and the mean SER of cases with non-tilted optic discs was − 1.17D ± 1.76D in the range of − 11 to 4D.

Training was performed for 50 epochs for each experiment, and a mini-batch of 16 was used. We achieved the best fine-tuning result with He initialization with normal, the Adam optimizer [42], a 1e-4 learning rate, and a 1e-3 learning decay rate [43, 44]. The dropout rate was set at 50% [45]. Categorical cross-entropy was used as a loss function for model training and validation. Our implementation incorporated the Keras and Tensorflow frameworks.

**Fig. 3:**
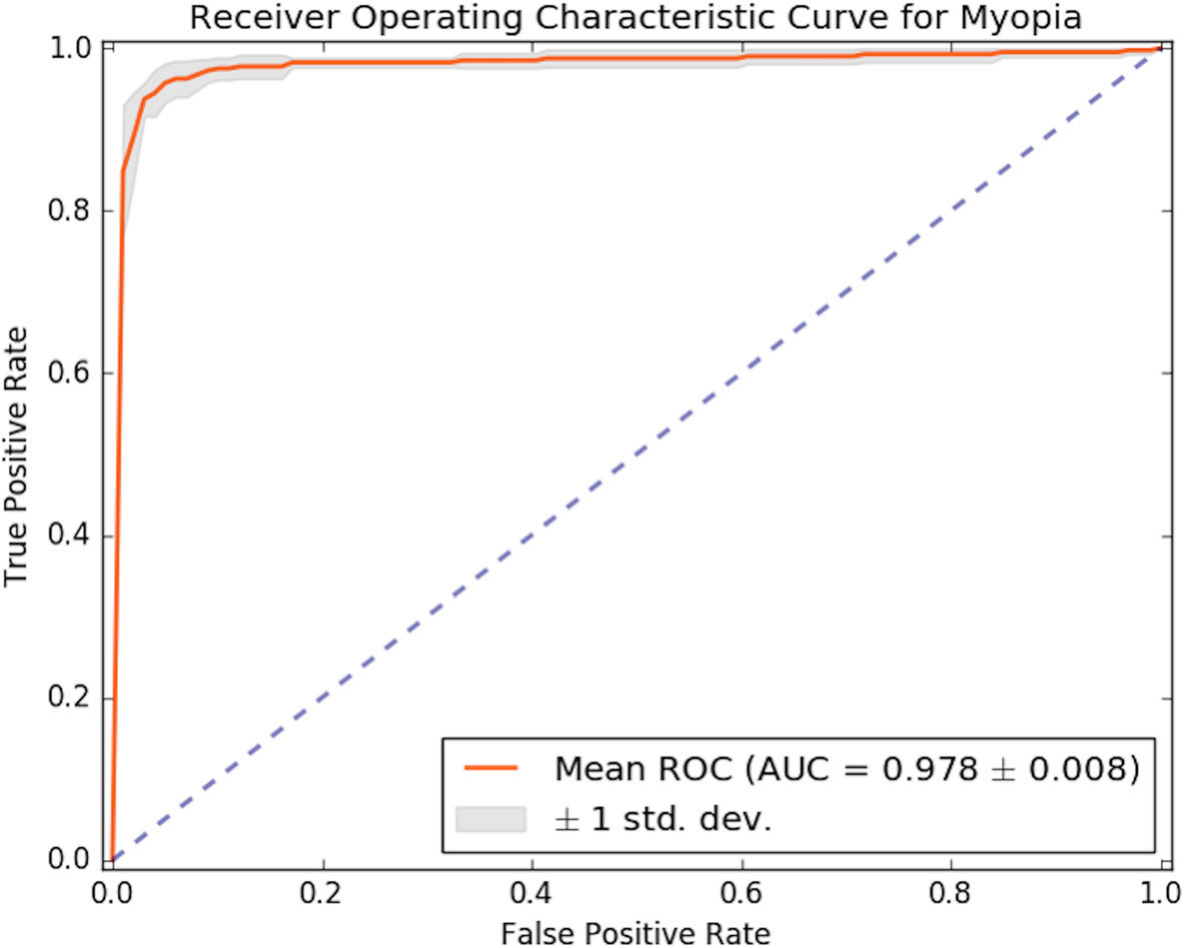
The mean receiver operating characteristic (ROC) curve derived from the stratified 5-fold cross-validation and the area under the curve (AUC) of the deep learning myopic tilted disc algorithm

Table 1 summarizes the performance of the deep learning model. Two aspect ratios were compared for each dataset to assess the simple resized dataset and the original AR-preserving dataset. Areas under receiver operating characteristic curves (AUCs) of the models using the simple image resizing (0.960 ± 0.017) were better than those that used preprocessing for original aspect ratio preservation (0.927 ± 0.083). The impact of augmentation was also evaluated. The AUCs were higher in the models that used 50 times the augmentation than in those that used the non-augmented dataset for both ARs. We found the best results when using the simple image resizing and augmentation, as follows: an AUC of 0.978 ± 0.008, an accuracy of 0.960 ± 0.010, sensitivity of 0.937 ± 0.023, and specificity of 0.963 ± 0.015. Figure 3 shows the mean receiver operating characteristic curve for the 5-fold cross-validation results of the best models.

**Table 1:** Cross validation results of the proposed models for myopic tilted discs

Dataset	Area Under the Curve	Average Validation Accuracy	Sensitivity	Specificity
AR Preserved	0.927 ± 0.083	0.910 ± 0.010	0.852 ± 0.082	0.916 ± 0.081
AR Preserved × 50	0.939 ± 0.010	0.921 ± 0.011	0.816 ± 0.054	0.935 ± 0.026
AR Distorted	0.960 ± 0.017	0.920 ± 0.013	0.867 ± 0.028	0.941 ± 0.015
AR Distorted × 50	0.978 ± 0.008	0.960 ± 0.010	0.937 ± 0.023	0.963 ± 0.015

**Fig. 4:**
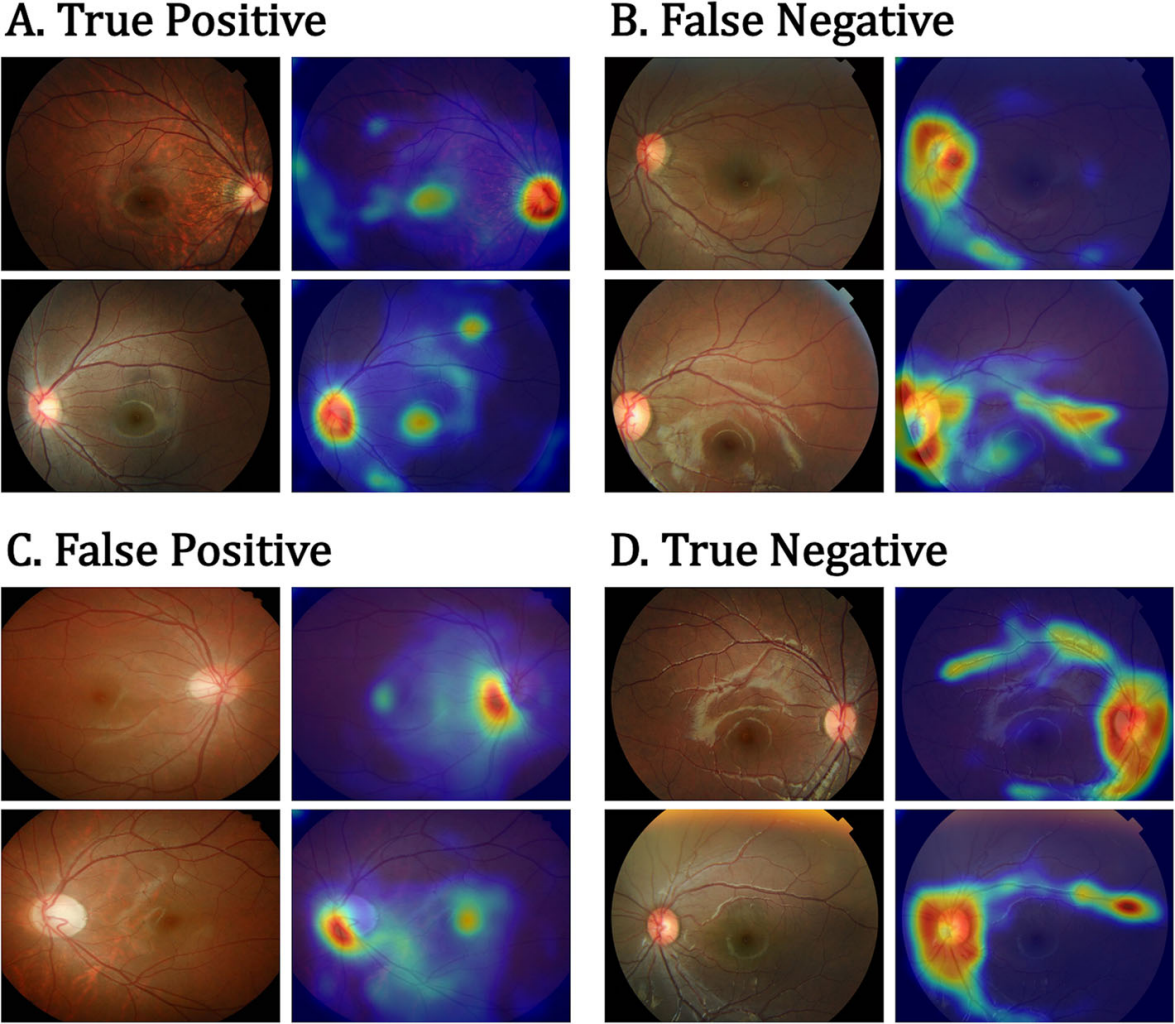
The confusion matrix of representative heat maps. **a** is correctly classified as myopic tilted disc images; **b** shows abnormal cases where the classifier was predicted as normal; **c** shows the opposite; **d** is correctly classified as normal optic disc images. Note that we used a threshold of 0.5 to generate the prediction outputs and heatmaps

Our model generates both a classification result based on the presence of the tilted optic disc and a heatmap that highlights the location of focus of the deep learning model. We used the Grad-CAM++ [46], which is a generalized visualization technique based on gradient-weighted class activation mapping [47], to generate the heatmaps. This is a powerful tool that can be used to identify visual features in input images that help interpret the results of the trained model. The generated heatmap is a single-channel image whose intensity values are normalized. The last convolutional layer from GoogleNet Inception-v3 was used to designate a gradient layer of the activation map. Figure 4 shows the Grad-CAM++ heatmaps and the corresponding original input images. When the AI model classifies an input image as an abnormal case, it focuses on the optic disc and retinal maculae, as illustrated in Fig. 4a (true positive) and c (false positive). In contrast, when it identifies an image to be a normal case, the heatmap highlights the wider area around the optic disc, often with superior retinal vascular arcades, as in Fig. 4b (false negative) and d (true negative). Accordingly, for images with the same prediction results, the model concentrated in similar areas and shapes to the heatmaps. However, it was difficult to understand why the model pays attention to those areas in the incorrectly classified cases.

## Discussion

In this study, we implemented and tested a deep learning approach to detect optic disc tilt using fundus photographs. We demonstrated that the proposed algorithm showed excellent agreement with the case definition of optic disc tilt in this study.

The algorithm showed reliable results for tilted optic disc classification (Table 1). Conventionally, the role and impact of image size have been emphasized, as has consistency in aspect ratio (AR) of a dataset maintaining intact shapes of objects of interest, which is crucial for accuracy [25]. In this study, we established a process to resize the dataset while preserving the original AR. However, the performance of the model showed unexpected results. Using the simple image resizing increased the scores compared with using the original AR preservation. Therefore, we proposed that preprocessing for original AR preservation might impact the deep learning model training for feature extraction, which further impacted the accuracy in a negative way.

Our deep learning approach exported the heatmaps to visualize the feature maps to generate outputs from the activation maps and determine the presence of tilted optic disc. In this study, Grad-CAM++ visualization revealed that the models were able to identify the location of the optic disc in most photographs, even though the models were trained without additional information about anatomical locations. The model seemed to not only highlighted the optic disc, but also traced significant retinal sections such as the superior retinal vascular arcade and the retinal macula. One interesting finding was that the heat map revealed difference between the two classes. The heatmap showed a ring-shaped region along the rim of the disc for cases predicted as negative (Fig. 4b and d), while it showed a round shape for cases classified as abnormal (Fig. 4 a and c). The model seemed to be focusing on the superior retinal vascular arcade for most of the abnormal cases even when the prediction was false. Meanwhile, the retinal macula was highly regarded in some of the cases with a positive prediction. Importantly, those retinal sections play a significant role in interpretation of the tilted optic disc by clinicians. Thus, we expect that heatmap visualization can help clinicians understand the result of the deep learning model [48]. However, interpretation of Grad-CAM++ visualization is subjective. We cannot entirely trust the heatmaps to locate anatomical findings. The primary goal was to demonstrate if the machine learning approach can distinguish myopic optic disc tilt in the fundus image. Our approach used weakly annotated data and tried to identify the visualized area used by the AI for the decision. Therefore, further research is needed to verify the relationship between interpretation results and actual ophthalmological anatomy. A segmentation-based future study will help provide clear interpretation.

Developing an algorithm that automatically discriminates disc tilt should precede development of an algorithm that corrects significant effects of disc tilt on ophthalmic instrumentation. Given the rapidly increasing myopic prevalence [3, 4], these types of algorithms can be integral parts of automated ophthalmologic diagnostic programs. Combined alteration of the optic nerve head in myopic optic disc tilt can vary and can include stretched vertical and/or horizontal dimension, with larger and shallower cups [49]. The degree of tilt and direction of disc tilt can also vary [49]. Nakazawa et al. reported one nasally tilted optic disc among 10 patients with mild or moderate myopia [22]. Although a peripapillary crescent developes gradually in the optic disc in most myopic patients [22], it is not a pathognomonic finding for myopic optic disc tilt. However, this study only considered temporally tilted discs with white semilunar patches of sclera adjacent to the optic discs as myopic tilted discs, and discs tilted in other directions were excluded. In addition, an optic disc with a ratio of minimal to maximal disc diameter of 0.75 or less was regarded as a definite tilted disc and was included in this study. As we mentioned earlier, appearance of the tilted disc may vary, and the definition varies among studies. It may be difficult to discriminate the excluded anatomical findings of this study from the myopic tilted disc using the developed algorithm. In photographs with false positives, the long to short axis ratio was greater than 0.75, but they had enlarged discs that deviated from the shape of the normal optic nerve head. In the study, based on the 0.75 long to short axis ratio, the cases where the optic nerves were ovoid and accompanied by slight peripapillary atrophy were included as tilted disc when visually discriminated. However, using the algorithms, they were excluded from the tilted disc. It may be that the algorithms detected the rotation of a three-dimensional disc more intrinsically than a deliberate research criterion. Further investigation of a wider range of myopic configurations of the optic disc using diverse devices such as optical coherence tomography is needed to enhance the accuracy and broader application of the program. Finally, a program that can analyze ophthalmic images and measurement values while correcting the effect of various degrees of optic disc tilt is needed for this patient population.

An ophthalmologic automatic diagnosis system can be of great help even for non-experts. For example, it can be useful in routine checkup or for non-tertiary hospitals. It might be effective and time-saving if an AI system could indicate or pre-select cases with non-tilted optic discs with a high confidence level as an automated screening tool. This research showed the possibility of AI-based automated tilted optic disc recognition. Since we already have trained a tilted optic disc detection model, we can train an advanced model with additional data for other anatomical changes as well as the excluded data by fine-tuning. We also can adopt various few-shot learning approaches [50] even if the amount of data is small. This could reduce the workload of ophthalmologists and non-experts. Also, further research could use the model that effectively employs a confidence level such as SelectiveNet [51].

There were several limitations to this study. First, we compared the accuracy of the algorithm with the results based on previous criteria of tilted optic disc [19, 20]. In the literature to date, optic disc tilt has been classified based on observations that are based on fundus photography [52–54]. Several studies have examined the optic nerve head of a patient with a myopic tilted disc using three-dimensional optical coherence tomography [52–54] or observed vascular abnormalities in a patient with a tilted disc using angiography [55, 56]. However, these previous approaches are not used as diagnostic standards. Therefore, because there are no accurate diagnostic criteria for using an objective device, this study used one of the previously published criteria that include an optic disc with a ratio of minimal to maximal disc diameter of 0.75 or less on the fundus photograph [19, 20] with a white semilunar patch of sclera adjacent to the optic disc. Second, we analyzed only temporally tilted discs, and it should be noted that the results of our study might not be valid when considering non-temporally tilted discs. Third, there may be limitations associated with using both eye data in this study due to possible inter-eye correlations. In future studies, the use of single eye data will be more desirable.

## Conclusions

In conclusion, we developed an automated system that detected optic disc tilt. The approach demonstrated excellent agreement with the previous clinical criteria which provides promising results for future programs that can help identify this condition. In addition, the approach for adjusting the effect of optic disc tilt on ophthalmic measurements can also be adapted, based on this novel approach.

## Data Availability

The datasets used in current study are available from the corresponding author upon reasonable request.

## Abbreviations

AUCs: areas under receiver operating characteristic curves
AR: aspect ratio
D: diopters
Grad-CAM: gradient-weighted class activation mapping
SER: spherical equivalent refraction
